# Current knowledge and interest of French Canadians regarding nutrigenetics

**DOI:** 10.1186/s12263-019-0629-7

**Published:** 2019-02-19

**Authors:** Bastien Vallée Marcotte, Hubert Cormier, Véronique Garneau, Julie Robitaille, Sophie Desroches, Marie-Claude Vohl

**Affiliations:** Institute of Nutrition and Functional Foods (INAF), Laval University, 2440 Hochelaga Blvd, Quebec, QC G1V 0A6 Canada

**Keywords:** Nutrigenetics, Personalised nutrition, Genetic testing, Public opinion, Attitudes, Dietitian

## Abstract

**Objective:**

The purpose of this study was to draw a global portrait of the current knowledge and interest regarding nutrigenetics in a population of French Canadians from the province of Quebec (Canada).

**Methods:**

A total of 2238 residents from the province of Quebec, Canada, were recruited via social networks and from the Laval University employee/student lists to participate in a 37-question online survey on nutrigenetics.

**Results:**

Most participants were not familiar with the term “nutrigenetics” (82.7%). Participants with good genetic literacy (26.8%) were less interested in nutrigenetic testing (*p* < 0.0001). The vast majority of participants (90.7%) reported to be willing to follow a personalised diet based on nutrigenetic testing, especially if they came to know themselves as carriers of a polymorphism increasing the risk of certain diseases. Participants had a higher interest in testing related to metabolic response to macronutrients (types of sugars, fats and proteins) than to micronutrients or other nutrients related to food intolerance.

**Conclusions:**

The attitude of French Canadians about nutrigenetics is very consistent with the results from other surveys published in the literature. Although few individuals are familiar with nutrigenetics, the public’s attitude towards nutrigenetics is globally favourable.

**Electronic supplementary material:**

The online version of this article (10.1186/s12263-019-0629-7) contains supplementary material, which is available to authorized users.

## Introduction

Nutrigenetics is defined by the role of DNA sequence variation in the responses to nutrients [1]. Advances in nutrigenetics have the potential to provide personalised nutritional recommendations through registered dietitians and thus could improve the efficacy of dietary interventions. In a non-diagnostic and preventive context, nutrigenetic tests can indicate which nutrients and foods could have beneficial effects on health while informing the individual about his/her future risks of developing certain long-term medical conditions such as cardiovascular diseases, inflammatory bowel diseases and even certain conditions falling within the sphere of bio-behaviour (depression, mood, psychological health) [2, 3]. These tests inform the individual about whether or not he/she is carrying a genetic variation that can either affect the metabolism of a particular nutrient or directly impact the consumption of various nutrients and/or foods.

Several companies specialised in nutrigenetics deliver the results of testing through the collaboration of a dietitian in order to guide dietary interventions (https://www.nutrigenomix.com). However, it has been reported in several studies that dietitians are not always familiar with nutrigenetics and do not consider themselves to be sufficiently qualified to use nutrigenetics in their professional practice, even though dietitians are considered to be the most reliable source of personalised nutrition information [4–6].

On the other hand, studies have shown that the population is generally interested in personalised nutrition via genetic testing [7–10]. Nielsen et al. found that patients are more likely to adhere to dietary recommendations if they are personalised according to their genetic profile [5]. Despite that few dietitians currently use it in their professional practice, the interest of the general population for personalised nutrition is increasing [9, 11]. Results from a qualitative study show that the population and health care professionals appear to have a poor understanding of nutrigenetics [12]. Global comprehension of the science of nutrigenetics as well as its potential beneficial outcomes on one’s health from both healthcare professionals and the population could be improved. In order to familiarise both dietitians and patients with DNA-based dietary advice and to facilitate its integration in professional practice, a prior evaluation of their current beliefs and perceptions towards this science is necessary.

Many studies in Europe, the USA and Canada attempted to determine the interest, acceptance, fears and perceived limitations of nutrigenetic testing and the use of their results in specific areas [5, 8, 12–17]. Due to rapid advances in the field of nutrigenetics, the population must be surveyed punctually in order to have the most updated data. Cultural, gender, social status and age differences are also important elements that need to be taken into consideration.

To date, no study has been conducted in French Canadians from the Province of Quebec to obtain their opinion regarding nutrigenetic testing, and the use of their results in a context of personalised nutrition. Consequently, the objective of this project was to evaluate the level of interest and current knowledge of nutrigenetics in the population of Quebec.

## Methods

### Proceedings

A total of 2238 residents from the province of Quebec (Canada), 18 years of age or older (mean age = 38.3 ± 14.9 years), were recruited via a social network (Facebook) and from the Laval University employee/student lists. Participants had to be able to answer the questionnaire written in French and to have access to a computer with an Internet connection. The invitation was sent on March 10, 2015, and the hyperlink was closed on April 28, 2015, at midnight. To reduce the risk that someone completes the survey twice (or more), the IP address of the computer used to complete the survey was checked. A total of 1535 individuals completed the survey and 110 individuals were excluded for not having answered properly to validation items, bringing the total to 1425 individuals (252 men (17.7%) and 1173 women (82.3%)).

### Questionnaire development

SurveyMonkey Gold with enhanced security (http://www.surveymonkey.com), an online survey development cloud-based software, was used to create the present study questionnaire. The questionnaire was pre-tested by 20 unrelated individuals to determine the necessary time to complete it and to attest the clarity of the questions and the relevance of the answer choices. The survey was made of 37 questions: 33 of them were closed-ended questions and 4 of them were open-ended questions. Most of the closed-ended questions were multichotomic with one or multiple possible answers, leaving the respondent the freedom to choose one or more of the answers (e.g. for personal and familial health history). Questions for quota sampling were also found at the beginning (i.e. citizenship, province or territory, administrative area and age) and at the end of the questionnaire (i.e. personal and familial health history, gender, ethnicity, matrimonial status, level of education, employment, field of study or work in addition to the previous year annual household income). Questions about citizenship, province/territory and the age were discriminatory to ensure that respondents were Canadian citizens living in the province of Quebec and were 18 years old or older. Genetic knowledge, also known as genetic literacy, has been evaluated using a 16-question questionnaire validated by Jallinoja and Aro, translated and validated in French [16, 18–20]. Each question was worth 1 point, for a maximum of 16 points. This 16-question questionnaire was included in the survey as one of the 37 questions, that is, 1 question of the questionnaire was composed of 16 sub-questions that participants had to answer by “true”, “false” or “I do not know”. An 11-point numeric rating scale (0–10) was also used to measure respondents’ level of interest to obtain DNA-based dietary advices specifically for 23 nutrients, including macronutrients and micronutrients in addition to some others such as caffeine, gluten, lactose, dietary fibre, alcohol or grains.

### Statistical analysis

Results were converted and downloaded into Excel (Microsoft, Redmonds, CA, USA) calculation sheets by SurveyMonkey and imported into SAS, version 9.3 (SAS Institute, Cary, NC, USA). Open-ended questions were compiled in a document and common themes have been identified using NVivo software v10.2.0. Results were analysed as either a continuous variable, ordinal variable or regrouped in quartiles. Literacy scores from the genetic knowledge questionnaire were grouped into quartiles as follows: < 10 (quartile 1), 10–11 (quartile 2), 12–13 (quartile 3), and 14–16 (quartile 4). Ordinal models for multinomial data adjusted for age and sex were used to assess the associations between genetic literacy and interest in nutrigenomics, and intention to adopt a personalised diet based on genetic tests results. Associations between categorical variables were assessed using a chi-square test. A *p* value < 0.05 was considered significant.

## Results

### Study population

Characteristics of subjects are shown in Table 1. The majority of respondents (82.3%) were women. The mean age was 38.3 ± 14.9 years. More than a quarter (25.2%) of the study participants had an annual household income of more than $100,000 CAD, and 49.4% had a university degree. Most of the participants were not familiar with the term “nutrigenetics” (82.7%). Individuals who were familiar with nutrigenetic testing had heard or read about it either in traditional media, such as television, newspapers and radio (27.7%), or from a dietitian (26.9%), web media (22.3%) or social network (14.6%). The least commonly cited sources were “social networks” (8.4%), “publicity” (3.8%) and “physician” (1.3%). In the present study sample, only five participants had previously undergone genetic testing.

**Table 1:** Characteristics of individuals who participated in the Quebec-wide e-consultation on nutrigenomics

	Total (*n* = 1425)	Men (*n* = 252)	Women (*n* = 1173)	*p* ^1^
Number (%)		17.7	82.3	
Age (years), *n* (%)				
18–29	537 (37.7)	60 (23.9)	477 (40.7)	0.0001
30–39	317 (22.3)	34 (13.6)	283 (24.1)	
40–49	197 (13.8)	47 (18.7)	150 (12.8)	
50–59	195 (13.7)	52 (20.7)	143 (12.2)	
60 and up	178 (12.5)	58 (23.1)	120 (10.2)	
Level of education, *n* (%)				
Elementary school	15 (1.1)	3 (0.2)	12 (0.8)	0.0001
High school/vocational training	150 (10.5)	19 (0.3)	131 (9.2)	
College	556 (39.0)	75 (5.3)	481 (33.8)	
University—undergraduate studies	364 (25.5)	49 (3.4)	315 (22.1)	
University—graduate studies	340 (23.9)	106 (7.4)	234 (16.4)	
Matrimonial status, *n* (%)				
Single	486 (34.1)	69 (27.4)	417 (35.6)	0.04
Married/common law	813 (57.1)	162 (62.3)	651 (55.5)	
Divorced/separated/widowed	109 (7.7)	20 (7.9)	89 (7.6)	
No answer	17 (1.2)	1 (0.4)	16 (1.4)	
Annual household income ($ CAD/year)				
≤ $19,000	138 (9.7)	15 (6.0)	123 (10.5)	0.0001
$20,000 to $39,999	140 (9.8)	20 (7.9)	120 (10.2)	
$40,000 to $59,999	211 (14.8)	26 (10.3)	185 (15.8)	
$60,000 to $79,999	173 (12.1)	33 (13.1)	140 (11.9)	
$80,000 to $99,999	198 (13.9)	30 (11.9)	168 (14.3)	
$100,000 and up	359 (25.2)	96 (38.1)	263 (22.4)	
No answer	206 (14.5)	32 (12.7)	174 (14.8)	
Urban centres, *n* (%)				
Quebec City	781 (54.8)	187 (74.2)	594 (50.6)	0.0001
Montreal	73 (5.1)	7 (2.3)	66 (5.6)	
Elsewhere in the province of Quebec	571 (40.1)	58 (23.0)	513 (43.7)	
Ethnicity, *n* (%)				
Caucasian	1378 (96.7)	244 (96.8)	1134 (96.7)	0.89
Others	46 (3.3)	8 (3.2)	39 (3.3)	

^1^Chi-square test was used to assess differences between subgroups

### Genetic literacy

**Fig. 1:**
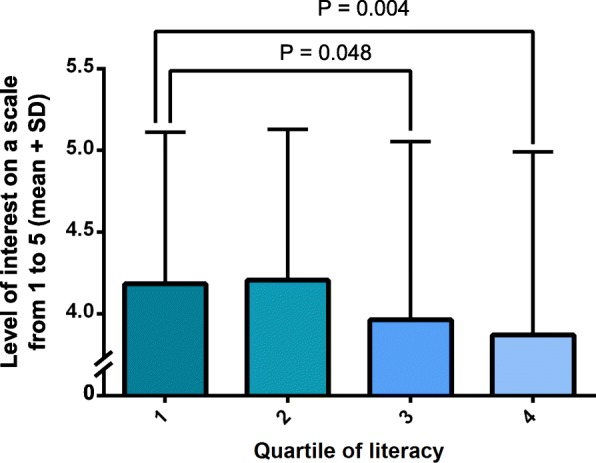
Level of interest in nutrigenetic testing according to quartiles of genetic literacy. SD is standard deviation

Genetic literacy was assessed using a validated 16-question questionnaire translated in French and included within the survey [18]. Globally, 3.6% (*n* = 51) of the participants had 16/16, followed by 9.0% of individuals who had 15/16 (*n* = 128). The mean score was 11.4 ± 2.8. When analysed as a continuous variable, genetic literacy was negatively associated with interest for nutrigenetic testing in an ordinal model for multinomial data adjusted for age and sex (*p* < 0.0007). When grouped into quartiles based on their genetic literacy score, individuals within the highest quartile (quartile 4) showed less interest for nutrigenetic testing compared to quartiles 1 (*p* = 0.004) and 2 (*p* = 0.0.001). Interest was also lower in quartile 3 compared to quartile 1 (*p* = 0.048) (Fig. 1). Will to follow personalised dietary advice based on genetic makeup was not different between quartiles, although a trend was observed between the second and the fourth quartiles (*p* = 0.053). Educational level was also inversely correlated with interest in nutrigenetic testing in a model adjusted for age and sex (Spearman partial correlation coefficient − 0.133, *p* < 0.0001).

### Personal or family medical history

There were associations with personal and/or familial medical history and the willingness to undergo a genetic testing. People were more inclined to follow a diet based on their genetic makeup if they had diagnosed hypertension (*p* = 0.03), diagnosed type 2 diabetes (*p* = 0.04), and personal obesity (*p* = 0.04) and if their parents had diagnosed type 2 diabetes (*p* = 0.01). The same association was observed if one of their grandparents was afflicted with inflammatory bowel disease (*p* = 0.04) or with cardiovascular diseases (*p* = 0.07).

### Nutrigenetic testing: what should be tested?

**Fig. 2:**
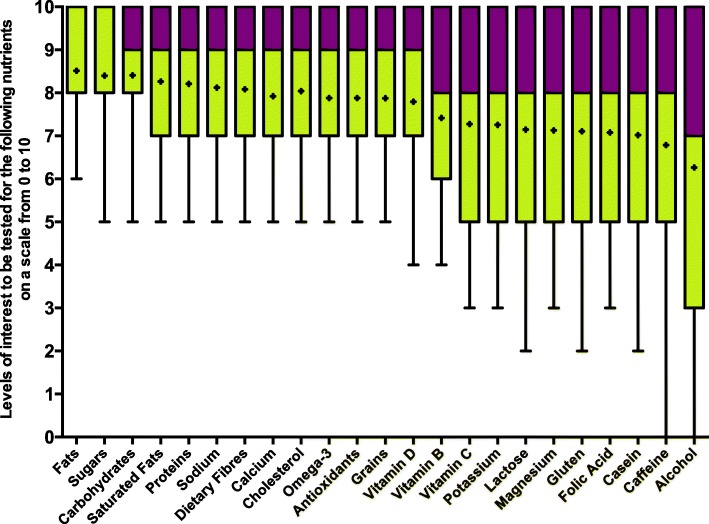
Box plot showing levels of interest to be tested for nutrients. The dots are the means, the bar separating the colours is the median, the bottom of the box is the 25th percentile and the whiskers are the minimum values

Participants were also asked to share their interest levels to be tested for 23 nutrients (fats, sugars, carbohydrates, saturated fats, proteins, sodium, dietary fibres, calcium, cholesterol, omega-3, antioxidants, grains, vitamin D, vitamin B, vitamin C, potassium, lactose, magnesium, gluten, folic acid, casein, caffeine and alcohol) on a numeric rating scale going from 0 to 10, where 10 was “extremely interested”. Mean scores for each nutrient are presented in Fig. 2. Briefly, participants had significantly higher interest levels to be tested for macronutrients such as fats, sugars, carbohydrates, saturated fats and proteins and lesser interest for alcohol and caffeine, and for other common nutrients associated with food intolerance such as gluten and lactose. By looking at the box plot, the interpretability, and the multiple comparison tests between each nutrient, five distinct clusters were identified as follows: (1) macronutrients (including fats, sugars, carbohydrates, saturated fats and proteins); (2) other nutrients commonly found on nutrition labels (sodium, dietary fibres, calcium, cholesterol, omega-3, antioxidants, grains, vitamin D, vitamin B and vitamin C); (3) minerals, nutrients associated with food intolerances and folic acid (potassium, lactose, magnesium, gluten, folic acid and casein); (4) caffeine; and (5) alcohol as two distinct clusters. Interest levels were similar for each component of a cluster, but varied from a cluster to another to such degree: macronutrients > other nutrients commonly found on nutrition labels > minerals, nutrients associated with food intolerances and folic acid > caffeine > alcohol (Additional file 1).

### Improvements in nutritional recommendations related to various diseases

We asked participants to which extent they would respect the following nutritional advice “Make the majority of your grain products whole grain each day” if they learned that they were carriers of a polymorphism in a gene responsible for an increased risk of type 2 diabetes. Eighty-five percent of participants answered “most likely” and “certainly” while only 1.7% answered “never” or “not likely”. Similarly, we asked them if they were inclined to respect the following dietary advice “make at least half of your grain products whole grain each day”, which is the current recommendation drawn from Canada’s Food Guide, knowing that they do not carry the genetic variation associated with a higher risk of type 2 diabetes. The percentage of participants that answered “most likely” and “certainly” dropped to 66.6% whereas 3.6% answered “never” or “not likely”.

## Discussion

This consultation aimed to better understand the current situation regarding knowledge and interest in nutrigenetics amongst French Canadians of the Province of Quebec in Canada. Motivations of this population to follow nutritional recommendations based on nutrigenetic tests results were documented. This survey had a response rate of 68.6%, which was considered acceptable [21, 22].

Expectedly, the majority of participants were not familiar with nutrigenetics, and most of the participants who had heard about nutrigenetic testing had either been informed via media or a dietitian. Kolor et al. also reported in an American study across four states that the most frequent sources by which individuals heard of genomic tests were television, radio, newspapers and magazines [23].

In this study, participants who had good genetic literacy were less interested in nutrigenetic testing. Morren et al. reported that a better genetic knowledge was associated with a more positive attitude towards genetic testing, and participants with a lower level of genetic knowledge had more difficulty to express an opinion about genetic testing [24]. In contrast, Poínhos et al. observed that individuals with perceived high levels of self-efficacy in nutrition had a more positive attitude towards personalised nutrition and were more prone to adopt personalised nutrition [14]. A possible explanation for this discrepancy is that individuals with good genetic literacy may deem nutrigenetic testing unnecessary for them to achieve healthy eating or could be more reluctant to undergo genetic testing, whereas individuals with poor genetic literacy may be more optimistic about the potential of nutrigenetics and could even overestimate its possible benefits. In this case, individuals’ interest in nutrigenetics could be following a certain Dunning-Kruger effect. The Dunning-Kruger effect can be defined as the illusion of knowing, or the observation that individuals who are unskilled tend to be unaware of their incompetency, and can therefore be more optimistic and manifest overconfidence when expressing their opinion about subjects they do not know [25–29]. As competency on a matter increases, the level of confidence tends to decrease because individuals realise their ignorance of the subject. Confidence is regained when a certain level of expertise is reached. It should be stressed here that participants with best genetic literacy are not experts in genetics either, and this could explain why they appear to have more conservative thoughts than participants with little knowledge. Consistently, an inverse correlation between educational level and interest in nutrigenetic testing was found.

It has been previously reported that individuals are more likely to adhere to dietary recommendations if they are based on their genetic profile [5]. In the present study, the vast majority of participants reported to be willing to adopt a personalised diet that is based on genetic testing. Moreover, more than 85% of participants reported to be ready to consume the majority of their grain products as whole grains if they were tested positive for an at-risk polymorphism for type 2 diabetes. This proportion decreased when participants knew they were not carrying the polymorphism. These findings further demonstrate that personalisation of dietary advice via nutrigenetics could constitute an important factor for the adherence to dietary recommendations. However, it also shows that nutrigenetics could be a double-edged sword. Participants appear to be highly motivated to change dietary habits if, according to their genetic profile, they are more at risk of developing a certain disease, but the opposite attitude could be observed when participants do not carry the at-risk polymorphism. In other words, individuals could possibly feel less concerned about the importance of healthy eating if they know that they have a “good” genetic makeup that does not predispose them to develop these diseases. For this reason, the implication of a health professional such as dietitians may help in the communication of nutrigenetic results to patients to favour a proper mindset towards nutrigenetics and avoid misinterpretations of results.

In the present study, participants were mostly interested in being tested for macronutrients rather than for micronutrients or nutrients associated with food intolerance. Individuals may perceive macronutrient intakes as more important determinants of health and weight management than other nutrients. It was previously reported that fat and sugar content of food was important in people’s perceptions of healthy eating [30]. Also, it was observed in a study that aimed to evaluate the public’s perceptions of a healthy diet that more than half of participants believed their intake of key nutrients for optimal nutrition was adequate through food [31]. Nonetheless, these results are rather surprising considering that self-reported food intolerance is on the rise and that it has become a trend that many individuals tend to avoid food containing compounds associated with food intolerance such as gluten [32, 33].

This study demonstrates that the overall perceptions, knowledge and attitudes of the French Canadian population regarding personalised nutrition via genetic testing are quite consistent with what has previously been reported in the literature with other populations. Although the public has a generally positive attitude towards nutrigenetics, very few are informed about its utilities and limits. This consultation will hopefully guide actions in order to adequately prepare and train health professionals, particularly dietitians, to integrate nutrigenetic tests into their professional practice.

## Additional file

Additional file 1: **Figure S1.** Levels of interest to be tested for the following nutrients on a scale from 0 to 10. (PDF 266 kb)
