# Supplementary figures and images for: Current knowledge and interest of French Canadians regarding nutrigenetics
Source: Genes Nutr. 2019 Feb 19;14:5. doi: 10.1186/s12263-019-0629-7 (PMC6380034; doi:10.1186/s12263-019-0629-7)

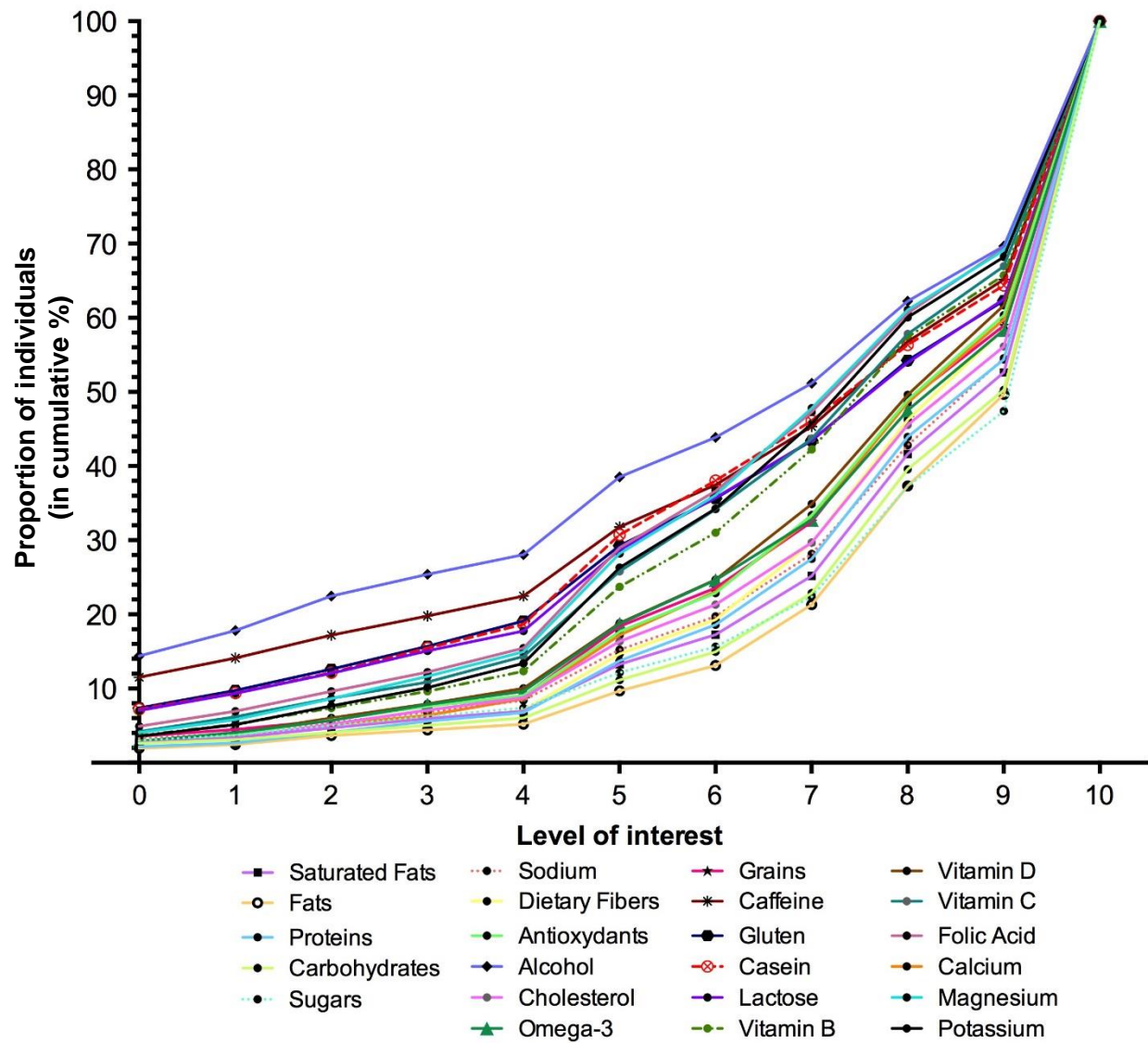

Supplement: Supplementary file 1 — Figure S1. Levels of interest to be tested for the following nutrients on a scale from 0 to 10. (PDF 266 kb) [file 12263_2019_629_MOESM1_ESM.pdf]
